# Mattel’s ©Barbie: Preventing Plasticizers Leakage in PVC Artworks and Design Objects through Film-Forming Solutions

**DOI:** 10.3390/polym16131888

**Published:** 2024-07-01

**Authors:** Andrea Macchia, Livia Marinelli, Francesca Irene Barbaccia, Tilde de Caro, Alice Hansen, Lisa Maria Schuberthan, Francesca Caterina Izzo, Valentina Pintus, Katiuscia Testa Chiari, Mauro Francesco La Russa

**Affiliations:** 1 Department of Biology, Ecology and Earth Sciences DIBEST, University of Calabria, Via Pietro Bucci, Arcavacata, 87036 Rende, Italy; andrea.macchia@unical.it (A.M.); mlarussa@unical.it (M.F.L.R.); 2 YOCOCU, Youth in Conservation of Cultural Heritage, Via T. Tasso 108, 00185 Rome, Italy; livia.marinelli@uniroma1.it (L.M.); lisaschuberthan@gmail.com (L.M.S.); katiuscia.testachiari@ied.edu (K.T.C.); 3 Department of Science of Antiquities, Sapienza University of Rome, Piazzale Aldo Moro 5, 00185 Rome, Italy; 4 Department of Technological Innovation Engineering, Digital Technologies for Industry 4.0, International Telematic University Uninettuno, Corso Vittorio Emanuele II 39, 00186 Rome, Italy; 5 CNR ISMN, Strada Provinciale 35d, 9, 00010 Rome, Italy; tilde.decaro@cnr.it; 6 Plart Museum, Via Giuseppe Martucci 48, 80121 Naples, Italy; a.hansen@plart.it; 7 Department of Environmental Sciences, Informatics and Statistics, Ca’ Foscari University of Venice, Via Torino 155, 30123 Venice, Italy; fra.izzo@unive.it; 8 Institute for Natural Science and Technology in Arts, Academy of Fine Arts Vienna, Schillerplatz 3, 1010 Vienna, Austria; 9 Institute for Conservation and Restoration, Academy of Fine Arts Vienna, Schillerplatz 3, 1010 Vienna, Austria

**Keywords:** p-PVC, phthalate, SEM analysis, microbial attack, chitosan, collagen

## Abstract

The main conservation problem of p-PVC artworks is phthalate-based plasticizer migration. Phthalate migration from the bulk to the surface of the materials leads to the formation of a glossy and oily film on the outer layers, ultimately reducing the flexibility of the material. This study aimed to develop a removable coating for the preservation of contemporary artworks and design objects made of plasticized polyvinyl chloride (p-PVC). Several coatings incorporating chitosan, collagen, and cellulose ethers were assessed as potential barriers to inhibiting plasticizer migration. Analytical techniques including optical microscopy (OM), ultraviolet/visible/near-infrared spectroscopy (UV/Vis/NIR), Fourier transform infrared spectroscopy with attenuated total reflection (FTIR-ATR), and scanning electron microscopy (SEM) were utilized to evaluate the optical and chemical stability of selected coating formulations applied to laboratory p-PVC sheet specimens. Subsequently, formulations were tested on a real tangible example of a design object, ©Barbie doll, characterized by the prevalent issue of plasticizer migration. Furthermore, the results obtained with the tested formulations were evaluated by a group of conservators using a tailored survey. Finally, a suitable coating formulation capable of safeguarding plastic substrates was suggested.

## 1. Introduction

The substantial advances in science during the 19th century can be credited with the introduction of almost 50 distinct polymer-based plastics into our daily life. Considered the first fully synthetic plastic, Bakelite (phenol formaldehyde) officially marked in 1907, the origins of plastics as we know them today, following earlier attempts that produced its semi-synthetic precursor (Parkesite). Synthetic plastics have proliferated due to their unique features, including workability, chemical stability, and electrical properties [1]. The impact of these man-made materials on industrial production is unquestionable: since their invention, science has given birth to different types of materials such as PVC (polyvinyl chloride), PP (polypropylene), PET (polyethylene terephthalate), and PS (polystyrene), just to mention some of them. This variety of materials, each with distinct properties and wide-ranging applications, has become a symbol of our most recent history and of the daily lives of modern society. Notably, plastics have charmed both scientists and artists, who have employed them as artistic mediums to convey innovative aesthetical and intangible concepts [2].

The toy industry has also embraced plastics. One of the most famous examples is the ©Barbie doll, which embodies the spirit of “plastic revolution” and the economic prosperity of the post-war era. Recent investigations have revealed that early ©Barbie dolls produced since 1959 were crafted from plasticized polyvinyl chloride (p-PVC). The composition of ©Barbie dolls evolved over the years due to the instability of polymer additives, resulting in the migration of plasticizers to the outer layers, and subsequent iterations gradually reduced the proportion of PVC in favor of other plastics. Consequently, the p-PVC surfaces of ©Barbie dolls used to exhibit a sticky appearance with oily exudates. Surface migration and plasticizer release also adversely affected the mass and internal stability of the polymer, impacting both material properties and human health [3,4,5]. The present study aimed to explore a solvent-removable coating suitable for application on p-PVC artworks to counteract plasticizer migration. 

According to the technical literature and cultural heritage standards, this experimentation considers the application of two materials: chitosan and collagen [6,7,8,9]. Indeed, the selected coatings meet the fundamental cultural heritage criteria (i.e., minimum intervention, compatibility) with a specific focus on reversibility as a critical feature. This principle is significant because it involves selecting coatings that may be applied and then removed without causing any changes to the original substrates. The evaluated materials must also meet additional conditions, such as not undergoing color changes or subsequent chemical reactions. Chitosan is a natural biopolymer with multiple potential applications [10,11]. It shows antimicrobial and antifungal properties, making it suitable for various purposes in the biomedical, industrial, and food packaging fields, especially for edible coatings [12,13,14]. Recent studies have highlighted chitosan’s effectiveness in the conservation of cultural artifacts. Interesting case studies have reported the successful use of chitosan nanoparticles, from the protection of paper-based artifacts to the preservation of historical building stones from biodeterioration [15]. Moreover, chitosan has proven to be valuable in preventing the growth of pathogenic fungal species in paintings [10]. Finally, chitosan coatings have demonstrated a promising ability to shield silver artifacts from oxidation and degradation [16]. 

Collagen, as well as chitosan, is developed as a material with barrier properties [17] capable of preserving the inherent characteristics of the internal product [18,19]. Collagen is employed in many applications, including food, biomedical, cosmetic, nutraceutical, pharmaceutical, and engineering industries [20,21]. Analytical methods have been developed to identify collagen-based materials originating from soft tissues and subsequently applied to diverse museum objects with successful outcomes [22]. Collagen has been employed as a consolidating agent in the conservation of cultural heritage artifacts [23]. The antimicrobial effects of peptide coatings, including collagen, have been investigated [24], and collagen’s potential in treating deteriorated skin has been demonstrated as well [25]. 

However, despite their interesting features, coatings based on chitosan and collagen are limited by poor mechanical and moisture barrier properties. Coatings composed of these substances tend to be inflexible and brittle [26,27]. Studies aimed at enhancing the mechanical properties of these substances have pointed out the role of cellulose fractions in enhancing chitosan’s mechanical properties, while cellulose derivatives have been found to improve collagen fiber aggregation [28]. Therefore, cellulose derivatives can remarkably modify and enhance collagen properties [29,30,31]. The aim of the research is to investigate the degradation mechanism and potential applicability of the coatings selected in this study in the cultural heritage field. As barrier coatings, capable of blocking the loss and subsequent continuous migration of the plasticizer from the bulk to the surface, non-toxic and removable formulations with non-toxic solvents for operators were chosen. Seven coatings have been tested as potential barriers against the leakage of plasticizers, starting from chitosan and collagen. Tests were conducted using optical techniques such as digital microscope and SEM, spectroscopy using FTIR-ATR, transmission spectroscopy, and spectrocolorimetry. Finally, the analysis of the oxygen transmission rate (OTR) was conducted.

## 2. Materials and Methods

According to the literature, the experimental strategy involved testing the decomposition of different chosen coating formulations made by combining chitosan and collagen. Pure coating-forming substances were also evaluated. Table 1 reports the details of the coatings used in the experimentation, both individually and in combination with each other.

Methylcellulose (Cuminal^TM^ MHPC 20000 S from Ashland, Covington, GA, USA) and hydroxyethyl cellulose (Natrosol^TM^ pharm HEC from Ashland, Covington, GA, USA) were employed to improve the application characteristics and spreadability, alongside natural rubber Latex (Prochima^®^, Metauro PU, Italy). To counteract the potential UV-induced degradation, a stabilizer (Tinuvin 292, BASF, Florham Park, NJ, USA) was introduced at a concentration of 0.5% in all formulations. The application of all formulations (5 mL) was carried out using a flat synthetic brush on a 0.2 mm p-PVC sheet provided by the Plart Museum (Naples, Italy) for the purpose of the study. It was prepared on a wooden frame and divided into 4 × 4 cm squares.

### 2.1. Laboratory Specimen Tests

The considered coatings for this study (Table 1), applied on transparent p-PVC substrates and left to dry for 24 h, were observed by visible light using an optical digital microscope Dino-Lite AM411-FVW, to define their morphology before and after aging.

The p-PVC specimens with applied coatings were investigated with different techniques before and after accelerated aging. The aim was to replicate the degradation observed in plastic materials. The aging process encompassed thermal degradation [38,39] and accelerated UV aging using a 340 nm UVA lamp, as described by Klempovà et al. [40]. Temperature cycles involved variations of 20 ± 1 °C [41] from 40 °C, while the relative humidity remained constant at 65% [38]. The analysis was conducted to identify any chromatic changes induced by the coatings on the surface and evaluate the coatings’ durability over time.

To define the potential variations induced by aging in film-forming substances, UV/Vis/NIR (ultraviolet/visible/near-infrared) transmission spectroscopy was employed. A light source emitting wavelengths between 300 and 1050 nm was passed through the p-PVC sheet, both with and without the different applied coatings. The amount of unabsorbed light was quantified using a MighTex USB Spectrometer HRS-BD1-025 (MighTex, Toronto, Canada) equipped with two bundles of optical fibers and a CCD Line Camera and a Toshiba TCD1304AP linear CCD array detector, with resolution 0.9 nm. The instrument’s light source operates within a wavelength range of 300–1050 nm, while the transmission spectra were collected between 400 and 1050 nm because a UV protector has been incorporated into the coating formulations. The probe head consisted of a homemade sample holder, black in color and measuring 4 cm on each side, with two specular openings (180° probe configuration). This configuration allowed for working without diffuse light and for collecting the incident light transmitted by the sample. To facilitate comparisons, spectral data underwent a standardization process: at each wavelength, the spectra of the substrate covered with the coatings (*Ii*) were subtracted from the spectrum of the p-PVC sheet (*I*_0_) using Equation (1):

(1) $$\Delta I=\sqrt{{\left(I\dot{i}-I_{0}\right)}^{2}}$$

The baseline is reported as dashed line, the spectrum of p-PVC was reported with error bar in the form of the standard deviation (SD) line, derived by computing the minimum and maximum values across all differences in the acquired spectra for each wavelength. This methodology allowed the evaluation of differences between the spectra of the substrate with the coatings and the spectrum of the substrate, highlighting the differences and their respective spectral regions. 

Spectrocolorimetric analysis was conducted using a Y3060 3nh spectrophotometer equipped with an 8 mm aperture lens and three Xenon light bulb, enabling simultaneous acquisition in SCI (specular component included) mode. Spectra were collected in the visible region (400–700 nm), under D65 CIE Standard illuminant, with the Standard observer at a 10° angle, employing an aperture mask with a diameter of 6 mm. Color was defined using CIE Lab color space coordinates L*, a*, b*, where L* indicates lightness, a* indicates variation in the redness-greenness range, and b* indicates variation in the blueness-yellowness range. Five measurements were taken for each tested area. Transparency of coating or color alteration were evaluated on the coating surface applied to a p-PVC sheet against a black background. The ΔE* parameter provided information regarding the total color shift of the surface. In accordance with the literature [42,43,44], the human eye can notice a barely noticeable difference in color between two objects only with ΔE* exceeds a value ranging between 2 and 3. For a better interpretation of trends in the visible for aged and unaged coatings and to define chromatic parameters, ΔE* was calculated using the formula commonly used in the cultural heritage sector, CIE 1976 (2) [45,46]:

ΔE* = [(ΔL*)^2^ + (Δa*)^2^ + (Δb*)^2^]½ where Δa* = a_2_ − a_1_; Δb* = b_2_ − b_1_; ΔL* = L_2_ − L_1_ (2)

To understand molecular changes that occurred in coatings, IR spectra were collected using FTIR (Fourier transform infrared) in the ATR (attenuated total reflection) mode. FTIR-ATR spectra were acquired using a Thermo Scientific Nicolet Summit FT-IR spectrometer (Thermo Fisher Scientific™, Waltham, MA, USA) equipped with Everest™ Diamond ATR accessory, which allows analysis in attenuated total reflectance (ATR) with a resolution of 4 cm^−1^ and a pressure device with 40 psi. A total of 32 scans were performed in each area. The acquired spectra were analyzed using the Library Manager, RRUFF™ Database, and scientific literature.

To assess the stability of the tested formulations against biological exposure, different coatings were contaminated with pathogenic agents from the *Aspergillus* family, commonly encountered in museum environments [47,48,49,50]. Mentioned coatings were incubated at 60 °C for three days. The stability against biological attack was evaluated using a Dino-Lite microscope, and ATP (Adenosine Triphosphate) levels were measured both before and after aging using a Lumitester PD 30 (Kikkoman).

Finally, the permeability of the coatings was analyzed according to the ASTM D1434-82 standard [51] and the OTR was measured with an N530 L Gas Permeability Analyzer (GBPI, testing instrument).

### 2.2. Application Test on a ©Barbie Doll as Case Study

The considered coatings were directly tested on a case study, represented by a severely degraded ©Barbie doll leg, as investigated by Macchia et al. [3]. 

This choice was prompted by the challenge of replicating comparable degradation levels in the laboratory. The p-PVC leg exhibited degradation characterized by the presence of phthalates on the surface [3], which had migrated from the bulk. This phenomenon resulted in an oily exudate attracting dust and environmental pollutants. Before the application of coatings, the surface was cleaned to remove exuded plasticizer, debris, dirt, and dust. This result was achieved using a binary solution (ligroin + ethanol mixture, 1:1) [52]. 

Six coatings (Film A, Film B, Film C, Film D, Film E, Film F, see Table 1) were sequentially applied from top to bottom of the cleaned surface to assess their performance characteristics on a case study object, which was subsequently subjected to artificial aging. Aging treatment was conducted using the same method employed for laboratory specimens. 

Optical digital microscopy (Dino-Lite) and scanning electron microscopic (SEM) analysis were employed to define the morphology of the applied coatings before and after aging. SEM analysis was conducted using SEM Tescam-VEGA 3 for potential coating degradation analysis at higher magnifications.

### 2.3. Applicability Survey 

A survey was carried out to define the applicability of selected coatings for the protection of plastic substrates. Although evaluation of the applicability of coating-forming film can be challenging, its consideration was crucial for the development of a formulation that was effectively suitable for practical use. Live workshops were organized to gather information about the ease of application and the potential removability of applied coatings using non-polar and non-toxic solvents such as water and ethanol. These activities allowed restorers and conservation professionals to express their opinions on the substances tested in the experiment. Workshops took place during the Plastics Heritage Congress at the Plart Museum in Naples in October 2022, and during the YOCOCU APS (Youth for Conservation of Cultural Heritage) Christmas Conference at the Museum für Kommunikation in Frankfurt, on December 8–9, 2022. A total of 85 participants attended the workshops, and they were asked to complete a survey (Table 2), providing their opinions and suggestions concerning application features, optical properties, and ease of coating removal for proposed coatings [53].

The first section of the survey (Part 1) was included to define restorers’ observations concerning the product before its application. Part 2 was conceived as the most significant section of the survey, in which restorers were asked to work with coatings and to provide feedback on the visual and physical properties that each individual coating shows during application. Part 3 provided insights into the satisfaction level of restorers with the coating, based on physical, visual, and processing time aspects. Finally, Part 4 addressed one of the goals of this study, which is the possibility of removing the coating with non-toxic solvents.

## 3. Results

### 3.1. Test on Laboratory Specimens

**Figure 1:**
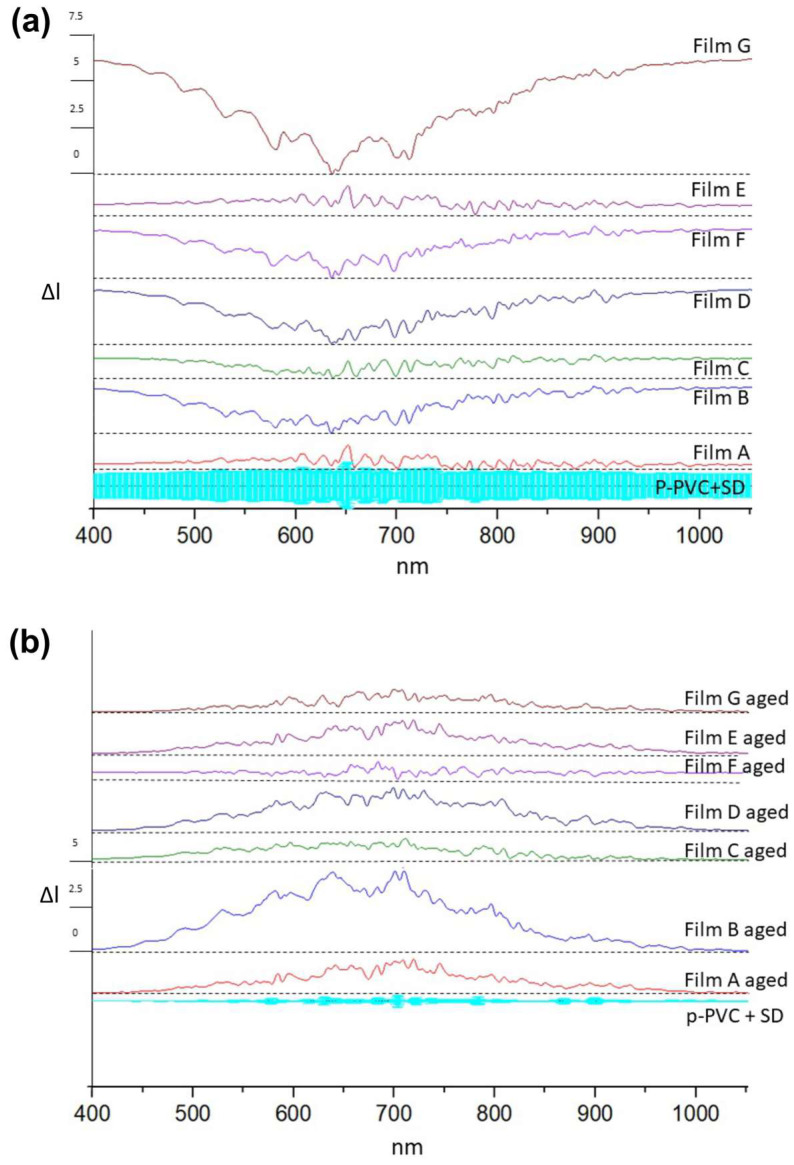
Transmission spectroscopy trends for both unaged (**a**) and aged (**b**) coatings subtracted from the p-PVC substrate spectrum, associated with standard deviation (light blue).

Figure 1a, b presents Vis/NIR spectral dispersions for both unaged and aged coatings in comparison to the substrate. 

Unaged Film G exhibited different spectra in comparison to the p-PVC substrate spectra, showing major absorption from 400 to 570 nm and after 730 nm. The Aged Film G spectra exhibited different spectra from the unaged one. The spectra of aged Film G were similar to the p-PVC substrate spectra. This change in visible light transmission could be attributed to changes in film morphology, like pore occurrence [54], and the transmission values could be attributable only to the p-PVC substrate transmission properties.

Unaged Film B exhibited transmission peaks in the region from 400 to 530 nm and after 720 nm. The spectra of aged Film B were different in comparison to the unaged spectra. Unaged Film D and F spectra showed similar variations occurring in Film B. After aging treatment, their light transmission properties varied from the initial ones. Film D shows a different transmission spectra to that of p-PVC, demonstrating that although it underwent modifications as a result of aging treatment, it resisted better than Films B and F. 

This suggests that in Film F, more variation occurred. Changes in absorption could be due to dehydration of film [41], in the case of Film D, or morphology change as previously reported as a consequence of the aging treatment. Films A, C, and E exhibited minimal variations compared to the substrate. All aged films presented transmission spectra near the substrate’s spectrum trend.

**Figure 2:**
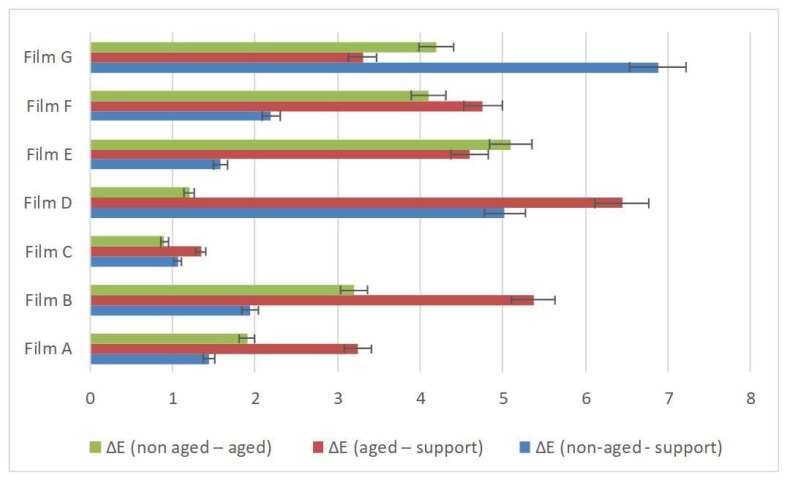
Results of spectrocolorimetric analysis of unaged, aging, and variation between the coatings. Blue color represents ΔE of unaged coatings on p-PVC sheet, orange color represents ΔE of aging coating on p-PVC sheet, and grey represents the confront of the ΔE variation between unaged and aging coatings.

Following the aging process, many materials underwent chemical and physical changes that may also induce chromatic variations. Spectrocolorimetric analysis can provide insights into this type of variation, even when changes are not visible to the human eye. Chromatic values are collected according to L*, a*, and b* parameters, and the resulting chromatic variation is expressed by parameter ΔE. In Figure 2, a comparison between ΔE values of unaged coatings and aged coatings is illustrated. Films B, D, E, F, and G presented alterations in chromatic parameters. L*, a*, and b* followed the aging process. This suggests a potential modification in the chemical composition or surface characteristics of these coatings. Significant alterations in chromatic aspects occurred, notably in component b*, while Films D and G shifted towards whitish-yellow tones. Conversely, Films B, E, and F varied towards bluish-white tones. On the other hand, Films A and C appeared to be chromatically stable even after aging, which indicates a good resistance to degradation or changes in surface properties.

FTIR-ATR spectra were acquired on coatings applied on p-PVC sheets to highlight the presence of the coating on the support and to detect any possible chemical changes induced by aging. The FTIR-ATR spectra were collected for both the unaged and aged coatings. A summary of peaks obtained from coatings is presented in Table 3, and a comparison between spectra of the aged and unaged coatings is shown in supplementary material. The collected spectra of the p-PVC substrate were defined by the following bands: 1718 cm^−1^ (C=O of phthalate ester) [55], 1405 cm^−1^ (CH_2_ bending) [3], and 1238 cm^−1^ (rocking of the C-H bonds) [55], 1116, 1088, and 1018 cm^−1^ (all within the C-O, C-O-H bond region) [56], and 950 cm^−1^ (aromatic C-H groups with out-of-plane vibration) [57,58]. Peaks at 852 and 660 cm^−1^ were attributed to C-Cl stretching [59].

The FTIR-ATR spectrum of Film A (Chitosan + Methylcellulose) in Figure S1 was characterized by typical bands of chitosan and methylcellulose as its main components used for formulating this type of coating: 1582 and 1426 cm^−1^ corresponded to asymmetric and symmetric vibration, respectively, of C=O of methylcellulose [60], while 1374 and 1320 cm^−1^ were related to the symmetric angular deformation of CH_3_ and the stretching of the CN- bond, typical of group III amines. Additionally, bands at 1156, 1075, 1033, and 956 cm^−1^ were attributed to C-O-C bond stretching [61,62,63,64], and the peak at 3362 cm^−1^ corresponded to the O-H group. Peaks in the 3700–3000 region were related to the O-H bond [65]. 

After the aging process, the spectrum exhibited enhanced definition of the bands between 1034 and 1075 cm^−1^, with a shift to 1075 cm^−1^ and a strong band at 1265 cm^−1^, related to characteristic CH_2_ wagging vibrations, combined N-H deformation, and C-N stretching [66]. The band at 1075 cm^−1^ was related to (C–O) generated by oxidation of carbons [67]. This was also supported by the peak at 1718 cm^−1^ attributed to the formation of ester group [68], and peaks at 1582 cm^−1^ and 1426 cm^−1^ confirmed the increase in absorbance of the C=O bond. This was evident from the observed increase in the absorbance ratio of 1030 cm^−1^/ 1580 cm^−1^ peaks in the spectrum of thermally treated film. 

The FTIR-ATR spectrum of Film B (chitosan + hydroxypropyl methylcellulose) in Figure S2 exhibited the following characteristic bands of chitosan: 3334 cm^−1^ (OH stretch), 1372 and 1315 cm^−1^ (CN- stretching involving N from the amino group III and CH_2_ vibration), 1062, 1023, and 948 cm^−1^ (peaks attributed to the C-O-C bond region) [34]. The spectrum collected after aging was characterized by the peaks of the p-PVC sheet at 1718 cm^−1^, the presence of peaks at 1278 and 1248 cm^−1^ in the rocking bond C-H region, and peaks at 1116, 1018, and 950 cm^−1^, along with the presence of weak-intensity bands from Film B. A peak at 3334 cm^−1^ was not present.

Spectra collected for Film C (collagen + methylcellulose) (Figure S3), both in its unaged and aged states, shared the same characteristic peaks: 3277 cm^−1^ (O-H bond), 1624, and 1524 cm^−1^ (N-H bending and C-N stretching of amides II and III) [35,69], 1396 and 1324 cm^−1^ (-OH vibration region) [64], 1240 cm^−1^ (amide III of type I collagen) [70], and 1058 and 1032 cm^−1^ (C-O-C and C-O bond) [71]. Only the degree of absorption changed slightly. 

FTIR-ATR spectra of unaged Film D (collagen + natural rubber) in Figure S4 were characterized by collagen bands, such as 1630 cm^−1^ (amide I associated with C=O stretching), 1547 cm^−1^ (amide II associated with N-H bending vibrations), and 1447 cm^−1^ (amide III group of collagens) [69,70]. Other IR bands can be observed, which arise because of natural rubber content. These are at 2959, 2927, and 2854 cm^−1^ (C-H stretching), 1660 cm^−1^ (C=C stretching), 1447 and 1375 cm^−1^ (C-H bending of natural rubber) [72,73]. The spectrum collected on aged Film D exhibited an absorption decrease in the regions of amines I, II, and III of the C=C and C-H bonds, in favor of a greater absorption of the C=O, OH, and C-OH bonds. The change may be due to the loss of C=C bonds in favor of the formation of C=O and C-OH bonds but not to the alteration of the amines [73]. The presence of the peak at 1718 cm^−1^ in aged Film D was associated with oxidative degradation [74]. 

Spectra collected on Film E (Methylcellulose) (Figure S5) were characterized by the following bands: 3334 cm^−1^ (O-H bond), 1320 cm^−1^ (CH_3_ groups), 1106, 1053 cm^−1^, 1028 cm^−1^, and 947 cm^−1^ (C-O-C bridge bond). After aging treatment, peaks at 1106 cm^−1^ and 1053 cm^−1^ shifted to 1110 cm^−1^ and 1059 cm^−1^, respectively, while peaks at 1718 cm^−1^ (ester group) and 1267 and 1250 cm^−1^ (C-H bond) appeared. In the spectra of aged Film E, the shifted peak at 1059 cm^−1^ increased in absorbance with respect to peak at 1053 cm^−1^ in the unaged Film E spectra, which also shifted. The peak at 1100 cm^−1^ increased in absorbance with respect to the peak at 1106 cm^−1^, and the peak at 1028 cm^−1^ disappeared on the aged Film E spectra. The peak at 3334 cm^−1^ shifted to 3437 cm^−1^.

Bands in unaged Film F (chitosan) (Figure S6) were similar to the bands observed in aged Film F. Peaks at 3334 cm^−1^ decreased and shifted to 3328 cm^−1^ (OH bond), but peaks at 1652 cm^−1^(C-N bond) [75] and 1374 and 1328 cm^−1^ (CN bond) remained. On the other hand, the peak at 1585 cm^−1^ (N-H bond) [75] disappeared, although the peaks at 1066, 1027, and 987 cm^−1^ (C-O bond) decreased in absorbance from unaged to aged Film F.

By comparing the spectrum of unaged Film G (hydroxypropyl methylcellulose) (Figure S7) with the aged one, it was possible to observe a shift of the peak at 1099 cm^−1^ (C-O-C bond) to 1101 cm^−1^. The peak at 1051 cm^−1^ decreased in intensity, in favor of a strong prominence of the peak at 1101 cm^−1^. The peak at 3334 cm^−1^ was seen in Film B to decrease in intensity and shift to 3362 cm^−1^. Moreover, the IR spectrum of the aged specimen was characterized by peaks at 1267 cm^−1^ and 1718 cm^−1^, related to the p-PVC substrate.

**Figure 3:**
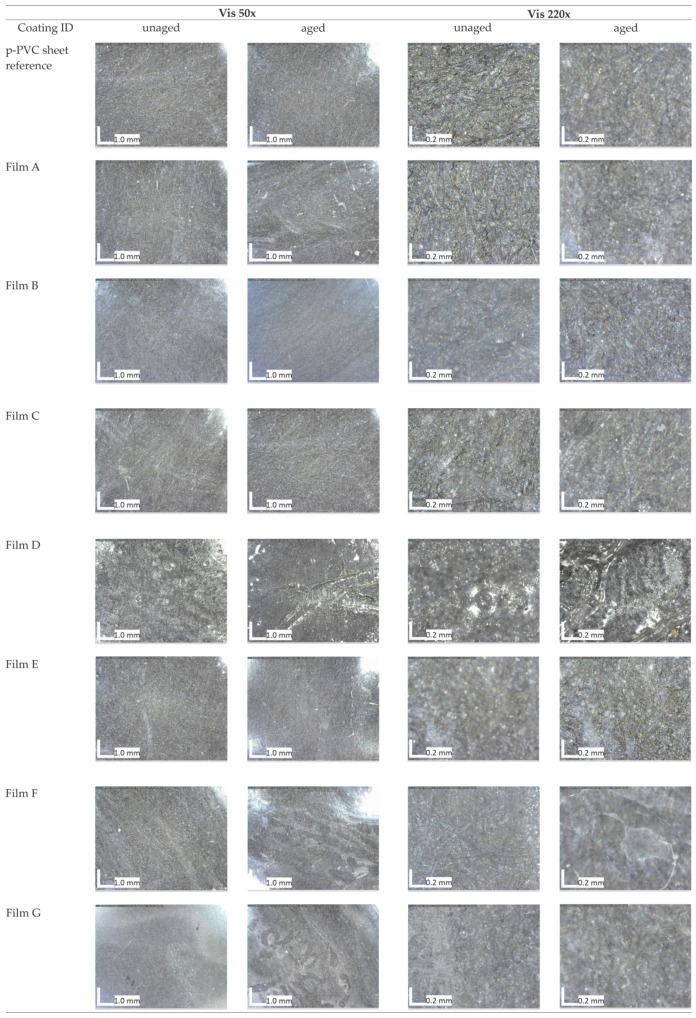
Digital microscope images of the coatings applied to the p-PVC sheet before, and after artificial accelerated aging.

Optical microscopic images collected on both unaged and aged films applied on p-PVC sheets are reported in Figure 3. Except for Films D and G, among the different unaged formulations, there were no significant alterations in the substrate’s appearance. The surface treated with Film G seemed opaque. Film D after drying resulted in an opaque-white aspect; this change was visible upon visual observation by optical microscopy analysis but was also supported by spectrocolorimetric results shown in Figure 2 and changes revealed in transmission spectroscopy and FTIR-ATR analysis. Moreover, comparing the results obtained from all analyses, namely spectrocolorimetry, transmission spectroscopy, FTIR-ATR, and optical microscope observations, it was observed that film D was the most susceptible formulation to the aging process. Observations under the microscope give us more information about the morphological changes of the coating, such as formation of bubbles, subsequent loss of adhesion of the substrate, and presence of fractures that we did not find with the same occurrence in other coatings. Finally, atmospheric particulate matter adhering to the surface of Film D could be observed at 220× magnification (Figure 3), in the shape of greyish pointy particles. Images from aged Films F and G revealed the presence of voids or cracks within the surface. On the other hand, Films A, C, and E did not exhibit substantial variations in their optical appearance and substrate cohesion.

**Figure 4:**
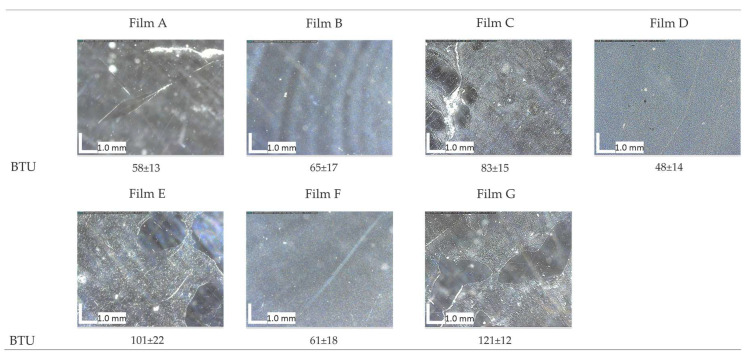
Optical microscope images after 72 h. Final ATP (adenosine triphosphate) values expressed in BTU units and reported for each film’s surface.

Resistance of the various formulations to *Aspergillus* biodeterioration was evaluated both morphologically by examining the surface using an optical microscope and by measuring the vitality of microorganisms in contact with the different formulations using a bio-luminometer. The luminescence of the microbial community was 4250 ± 67 BTU (Bio-treatment unit). Optical microscope images (Figure 4) collected after 72 h did not reveal any morphological changes in the films, and BTU values were close to zero, confirming that the surfaces did not provide a suitable substrate for microbiological proliferation.

The obtained values for the oxygen transmission rate (OTR) are shown in Table 4. The permeability of oxygen was not lower for composite film and increased for the composite one. The transfer was favorable to the presence of hydrophobic groups induced while decreasing with polar groups, as for Film B. For this reason, the obtained values were close for all tested coatings.

### 3.2. Test on ©Barbie Doll

Summarizing the results obtained from optical, colorimetric, and spectroscopic analysis, as well as keeping in mind the results of the biological attack and the opinions collected from the test with restorers, we identified Films A, C, E, and F as the coatings showing a better resistance to aging, both from a point of view of color alteration and from a chemical point of view. Films B, D, and G, on the contrary, showed alterations in color or alterations from a chemical point of view. Coatings must not induce chromatic alterations on the surface and must exhibit a strong adhesion to the surface, which establishes interactions between the molecules of the applied coating and the substrate’s molecules.

Optical microscopy and SEM were used to evaluate the morphology of several coatings on the ©Barbie leg’s surface, before and after aging. 

**Figure 5:**
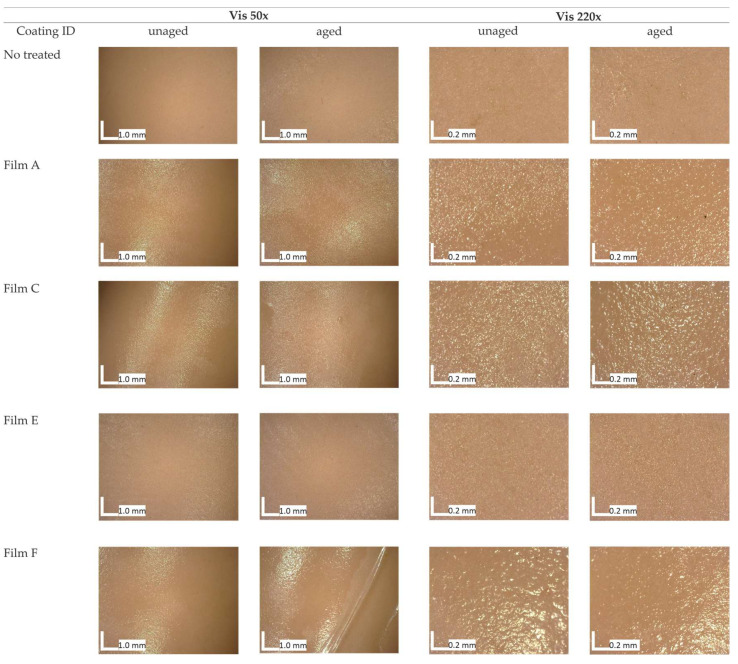
Digital microscopy images from visible light before and after aging treatment on the non-treated surface of the ©Barbie leg.

Images captured through optical microscopy (Figure 5) on unaged coatings highlighted their potential to cover the ©Barbie’s leg surface homogenously. Film E was smooth and opaque, while films A, F, and C appeared moderately rough in comparison with the untreated surface. The roughness of Films A and C increased with the aging process, while Film F detached from the surface. This result highlighted imperfect adhesion between the film based on chitosan only and the surface.

**Figure 6:**
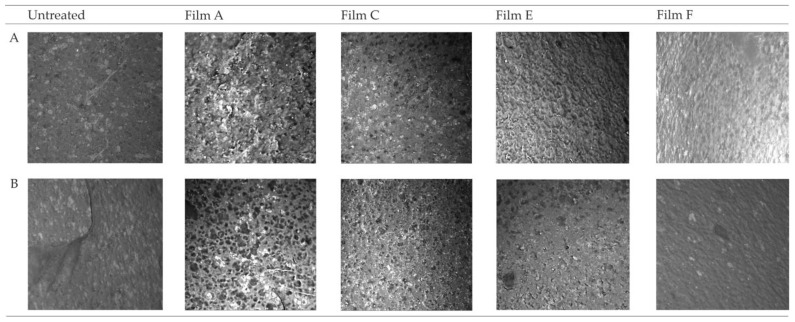
SEM images of the Films applied on the surface of the ©Barbie leg before (**A**) and after (**B**) aging.

Images collected through SEM (Figure 6) revealed in Films A and C, the formation of composite structures where cellulose fibers were dispersed in the organic matrix, while in Films E and F, a homogeneous layer formed on the surface. This result defined a rougher surface morphology for Films A and C in comparison to E and F. Following the aging process, the films were altered by the crosslinking process, which promoted in Film A > Film C, the increasing roughness with the formation of aggregation due to the shrinkage of the film. For Films E and F, there was non-adhesion between the film layer and the substrate, where they seemed to have disappeared, proving that the critical issue for the application of collagen and chitosan is the stability over time. The phenomenon found in Films A and C could be associated with a Voronoi tessellations phenomenon due to the evaporation of interstitial water in the methylcellulose [76].

### 3.3. Survey 

The ease of application, although difficult to scientifically evaluate, constitutes a crucial factor in comprehending the practical utility of coating within the realm of cultural heritage. Table 5 reports the results of the survey taken by restorers. 

Based on an optical observation of the coating, restorers agreed on defining all formulations as transparent, except in the case of Film D, which was opaque. In addition, restorers agreed that Films A, B, E, and G were viscous, Films D and F were liquid, and Film C was defined as fluid. In Part 2, the film with best ratings was Film C. Its fluid consistency was considered easy to work with during application, with the tendency to separate low that forms a more uniform texture compared to other coatings. In Part 3, the films observed to have the fastest evaporation rate, showing a transparent appearance, and feeling smooth to the touch as the substrate surface were Films C, F, and G. In the last section (dedicated to the removal), all films were considered easy to remove using water or an alcohol-water solution. The most adequate films from a technical point of view, however, were Films B, C, F, and G, having a degree of removal with the use of water judged to be medium; consequently, it was little subject to environmental humidity.

## 4. Discussion

Coatings developed using single component pure substance such as Films E and F exhibited superior morphological characteristics when compared to composite materials (Film A, B, and C). Optical microscope images revealed a rougher surface in the case of the composite materials, especially when cellulose was used as an additive (Film A). The addition of methylcellulose provided an enhanced resistance to degradation. This result is evident in the differential behavior observed between Film A and Film B during the aging process. Surface variations observed on the p-PVC sheet treated with Film B after aging could be attributed to the higher solubility of hydroxypropyl methylcellulose compared to methylcellulose, leading to solubilization and contraction induced by aging. The FTIR-ATR spectra obtained from Film D defined a chemical degradation induced by reticular oxidation. The phenomenon that may have induced the detachment and loss of material in Films B, D, and G could be related to the dewetting phenomenon induced by the loss of small water accumulations within the film structure [77,78]. The contraction found in the films, if attributed to a dewetting phenomenon, is reflected in the decrease in absorption intensity in the FTIR-ATR peaks relative to the hydroxyl group. Films E and G did not yield suitable results for the purposes of this study due to their alteration of the chromatic aspect. This alteration was noticeable on results obtained from both spectrocolorimetric and UV/Vis/NIR spectroscopy techniques, where it was possible to notice a variation on the aged and unaged film spectra confirmed by the variation in the spectrocolorimetric results. SEM analysis of Film A revealed the formation of a disordered network between the coating components. The SEM image of aged Film A revealed the contraction of the chitosan matrix, a conclusion corroborated by the observation with optical microscopy of the ©Barbie’s surface, where the appearance became more similar to the untreated leg. The survey completed by the conservators highlighted difficulties in applying Films D and F, while Films C and E proved to be easier to work with. All tested films could be easily removed with ethanol, except for Film D. Notably, Film B exhibited a glossy appearance upon drying, unlike the transparency of the other films. Regarding oxygen permeability, all materials aligned with the results in the existing literature. Among the investigated coatings, Film C exhibited the most suitable characteristics for the application on the surfaces considered in this study: it displayed excellent resistance to aging when compared to other formulated films; it possessed surface morphological properties that provided a visual appearance considered compatible with the opaque p-PVC substrate of the ©Barbie doll under study; and it demonstrated an ease of application on both smooth and rough p-PVC surfaces. Future research may explore other application methods aimed at investigating the characteristics and potential applications of the film substances produced in this study.

## 5. Conclusions

This study investigated the feasibility of the application of the coatings selected from the existing literature to address the specific challenge of creating a barrier against plasticizer migration in p-PVC-based cultural heritage. This research explored the impact of some coatings on a p-PVC sheet plasticized with phthalate. The assessment involved the examination of laboratory samples before and after accelerated artificial aging using optical microscopy, SEM, UV/Vis/NIR, color measurements, and FTIR-ATR.

The absence of physical and chemical variations after aging is an important issue for employing the film in the treatment of cultural heritage. The most promising film among those tested was subsequently applied on the leg of the ©Barbie, to test the performance on a real case, with the aim of preserving the degraded doll. The result of coating application was studied using optical microscopy and SEM analysis, enabling us to conclude that the most suitable coating for forming a protective film was Film C: collagen and methylcellulose. This finding was further validated through a hands-on experiment that engaged different restorers who shared their valuable subjective experiences via a specialized survey. Because dewetting processes in coatings can occur, it is advisable to limit the use of these coatings to environments with appropriate thermo-hygrometric quality in museums.

## Figures and Tables

**Table 1:** Composition of the selected coating-forming film and the preparation procedures.

Coating ID	Composition	Preparation	References
Film A	Chitosan + Methylcellulose	1.5 g chitosan in 100 mL of 1% acetic acid aqueous solution. To adjust the pH value into more neutral conditions, 1 M NaOH was added until pH 6.2 was obtained. 1.5 g methylcellulose dissolved in 50 mL of 50% ethanol solution. Chitosan and methylcellulose were mixed under stirring.	[32,33]
Film B	Chitosan + Hydroxypropyl Methylcellulose	Composite coating is prepared from chitosan-HPMC 50:50 proportionally. Chitosan is dissolved in 1% acetic acid aqueous solution to prepare a 2% w/v chitosan aqueous solution. 9 parts of HPMC are dissolved in distilled water (200 parts) and ethanol (100 parts).	[34]
Film C	Collagen + Methylcellulose	15 mg/mL collagen is dissolved in 0.1 mol/mL acetic acid aqueous solution and then added 15 mg/mL HPMC solubilized in 50:50 water ethanol solution	[35]
Film D	Collagen + Natural rubber	7.5 g of collagen powder is solubilized in 50 mL distilled water and then added 0.04 mL of natural rubber ready to use.	[36]
Film E	Methylcellulose	Methylcellulose 3% w/v is mixed with water-ethanol solution 50:50 v/v.	[37]
Film F	Chitosan	1.5 g chitosan in 100 mL 1% acetic acid aqueous solution chitosan w/v in 1% acid acetic + 0.2 g glycerol	[37]
Film G	Hydroxyethyl Cellulose (HEC)	HPMC is dissolved in solution water-ethanol as in preparation of Film C	[34]

**Table 2:** Survey submitted to workshop participants.

Part 1—Analysis of the coating before the application		
Appearance: - Gloss - Opaque/Mat - Transparent	Consistency: - Liquid - Fluid - Thick - Viscous	
Part 2—Analysis of the coating during the application		
Appearance: - Gloss - Opaque / Mat - Transparent	Consistency: - Liquid - Fluid - Thick - Viscous	Separation tendency of the Coating into different phases: - High—unstable - Medium—moderately unstable - Low—stable
Application on the surface: - Easy - Medium - Difficult	Texture: - Uniform - Non-uniform	
Part 3—Evaluation after the formation of the coating		
Appearance: - Gloss - Opaque / Mat - Transparent	Drying rate: - High - Medium - Slow	Touch sensation: - Sticky - Gummy - Smooth - Rough
Part 4—Coating removal		
Mechanical action: - Easy - Difficult - Ineffective	Solvent action: 1. Deionized water - Easy - Difficult - Ineffective	2. Ethanol - Easy - Difficult - Ineffective
Coating surface wettability: - Bad - Medium - Good	Removal rate: - Slow - Medium - Fast	Need to apply poultice to speed up the removal: - Y - N
Effects of removal on the surface: - None - Mild - Consistent		

**Table 3:** Summary of FTIR-ATR peaks.

	Unaged		Aged	
ID Sample	Wavenumber (cm^−1^)	Molecular Bond	Wavenumber (cm^−1^)	Molecular Bond
PVC substrate	1718 1405 1238 1116, 1088, 1018 950 852, 660	C=O ester CH_2_ bending C-H rocking C-O and C-O-H C-H aromatic vibration C-Cl stretching		
Film A	3362 1582, 1426 1374, 1320 1156, 1075, 1033, 956	O-H stretch C=O asymmetric and symmetric vibration CH_3_ symmetric angular deformation C-O-C bond stretching	3328 1718 1582, 1425 1075, 1034, 956	O-H stretch C=O ester C=O CH_3_
Film B	3334 1372, 1315 1062, 1023, 948	O-H stretch CN- stretching involving N from amino group III and CH_2_ vibration C-O-C	1718 1278, 1248 1116, 1101 1018 950	C=O ester C-H rocking bond C-O C-O-H C-H
Film C	3277 1624, 1524, 1240 1445 1396, 1324, 1240 1058; 1032	O-H stretch N-H bending and C-N stretching of amides II and III C-H bending OH vibration Amide III of type I collagen C-O-C and C-O	3278 1624, 1524 1445 1396, 1324, 1240 1058; 1032	O-H stretch N-H bending and C-N stretching of amides II and III C-H bending OH vibration Amide III of type I collagen C-O-C and C-O
Film D	1660, 1630 1547 1447 2959, 2927, 2854 1447, 1375 1324, 1243 1063, 1017	amide I associated with C=O N-H bending amide III of collagen C=C stretching C-H bending OH vibration C-O-C	1718 1447, 1375 1266; 1249 1115, 1101, 1018	C=O ester amide III of collagen C-O C-O-C
Film E	3334 1320 1106, 1053, 1028, 947	O-H stretch CH_3_ C-O-C	3437 1718 1267, 1250 1106, 1059	O-H stretch C=O ester C-H C-O-C
Film F	3334 1652 1585 1374, 1328 1066, 1027, 987	O-H stretch C-N N-H CN C-O	3328 1652 1374, 1328 1066, 1027, 987	O-H stretch C-N CN C-O
Film G	3334 1099, 1051, 943	O-H stretch C-O-C	3362 1718 1267 1101	O-H stretch C=O ester C-H C-O-C

**Table 4:** Oxygen permeability values of the tested coating.

	OTR (cm^3^/m^2^)	SD
Film A	0.48	0.005
Film B	0.29	0.032
Film C	0.39	0.012
Film D	0.29	0.003
Film E	0.15	0.012
Film F	0.34	0.007
Film G	0.22	0.003

**Table 5:** Assessment pertaining to the performance of diverse films derived from interviews conducted with restorers.

		Film A	Film B	Film C	Film D	Film E	Film F	Film G
**Part 1**	Appearance	Transparent	Transparent	Transparent	Opaque / Mat	Transparent	Transparent	Transparent
	Consistency	Viscous	Viscous	Fluid	Liquid	Viscous	Liquid	Viscous
**Part 2**	Appearance	Gloss	Gloss	Gloss	Opaque / Mat and Gloss	Gloss	Gloss	Gloss
	Consistency	Viscous	Viscous	Fluid	Liquid	Viscous	Liquid	Viscous
	Separation tendency	Low	Low	Low	High	Low	Medium	Low
	Texture	Non-uniform	Non-uniform	Uniform	Non-uniform	Uniform	Uniform	Non-uniform
	Application	Medium	Medium	Easy	Difficult	Easy	Difficult	Easy
**Part 3**	Appearance	Transparent	Opaque / Mat	Transparent	Gloss	Transparent	Transparent	Transparent
	Drying rate	Medium	High	High	Medium	Medium	High	High
	Touch sensation	Smooth	Smooth	Smooth	Gummy	Smooth	Smooth	Smooth
**Part 4**	Mechanical cleaning	Ineffective	Ineffective	Ineffective	Easy	Ineffective	Ineffective	Ineffective
	Deionized water-based cleaning	Easy	Difficult	Difficult	Ineffective	Easy	Difficult	Difficult
	Ethanol -based cleaning	Easy	Easy	Easy	Difficult	Easy	Easy	Easy
	Wettability	Good	Medium	Medium	Bad	Good	Medium	Medium
	Removal rate	Fast	Medium	Slow	-	Fast	Medium	Medium
	Need poultice	No	No	No	No	No	No	No
	Effect on surface	None	None	None	None	None	None	None

## Data Availability

The original contributions presented in the study are included in the article/Supplementary Materials, further inquiries can be directed to the corresponding author.

## Supplementary Materials

The following supporting information can be downloaded at: https://www.mdpi.com/article/10.3390/polym16131888/s1, Figure S1: Comparison of FTIR-ATR spectra collected from Film A unaged and aged, both applied on the p-PVC sheet, Film A as a single component, and p-PVC sheet; Figure S2: Comparison of FTIR-ATR spectra collected from Film B unaged and aged, both applied on the p-PVC sheet, Film B as a single component, and p-PVC sheet; figure S3: Comparison of FTIR-ATR spectra collected from Film C unaged and aged, both applied on the p-PVC sheet, Film C as a single component, and p-PVC sheet; Figure S4: Comparison of FTIR-ATR spectra collected from Film D unaged and aged, both applied on the p-PVC sheet, Film D as a single component, and p-PVC sheet; Figure S5: Comparison of FTIR-ATR spectra collected from Film E unaged and aged, both applied on the p-PVC sheet, Film E as a single component, and p-PVC sheet; Figure S6: Comparison of FTIR-ATR spectra collected from Film F unaged and aged, both applied on the p-PVC sheet, Film F as a single component, and p-PVC sheet; Figure S7: Comparison of FTIR-ATR spectra collected from Film G unaged and aged, both applied on the p-PVC sheet, Film G as a single component, and p-PVC sheet.
