# Supplementary material for: Mattel’s ©Barbie: Preventing Plasticizers Leakage in PVC Artworks and Design Objects through Film-Forming Solutions
Source: Polymers (Basel). 2024 Jul 1;16(13):1888. doi: 10.3390/polym16131888 (PMC11244241; doi:10.3390/polym16131888)
Supplement: Supplementary file 1 [file polymers-16-01888-s001.zip › polymers-3032722-supplementary.pdf]

polymers

MDPI

Article

Mattel's ©Barbie: Preventing Plasticizers Leakage in PVC Artworks and Design Objects through Film-Forming Solutions

Andrea Macchia [1,2,†], Livia Marinelli [2,3], Francesca Irene Barbaccia [2,4,†], Tilde de Caro [5], Alice Hansen [6], Lisa Maria Schuberthan [2], Francesca Caterina Izzo [7], Valentina Pintus [8,9,*], Katiuscia Testa Chiari [2] and Mauro Francesco La Russa [1]

1 Department of Biology, Ecology and Earth Sciences DIBEST, University of Calabria, Via Pietro Bucci, Arcavacata, 87036 Rende, Italy
2 YOCOCU, Youth in Conservation of Cultural Heritage, Via T. Tasso 108, 00185 Rome, Italy
3 Department of Science of Antiquities, Sapienza University of Rome, Piazzale Aldo Moro 5, 00185 Rome, Italy
4 Department of Technological Innovation Engineering, Digital Technologies for Industry 4.0, International Telematic University Uninettuno, Corso Vittorio Emanuele II 39, 00186 Rome, Italy
5 CNR ISMN, Strada Provinciale 35d, 9, 00010 Rome, Italy
6 Plart Museum, Via Giuseppe Martucci 48, 80121, Naples, Italy
7 Department of Environmental Sciences, Informatics and Statistics, Ca' Foscari University of Venice, Via Torino 155, 30123 Venice, Italy
8 Institute for Natural Science and Technology in Arts, Academy of Fine Arts Vienna, Schillerplatz 3, 1010 Vienna, Austria
9 Institute for Conservation and Restoration, Academy of Fine Arts Vienna, Schillerplatz 3, 1010 Vienna, Austria
* Correspondence: v.pintus@akbild.ac.at
† These authors contributed equally to this work.

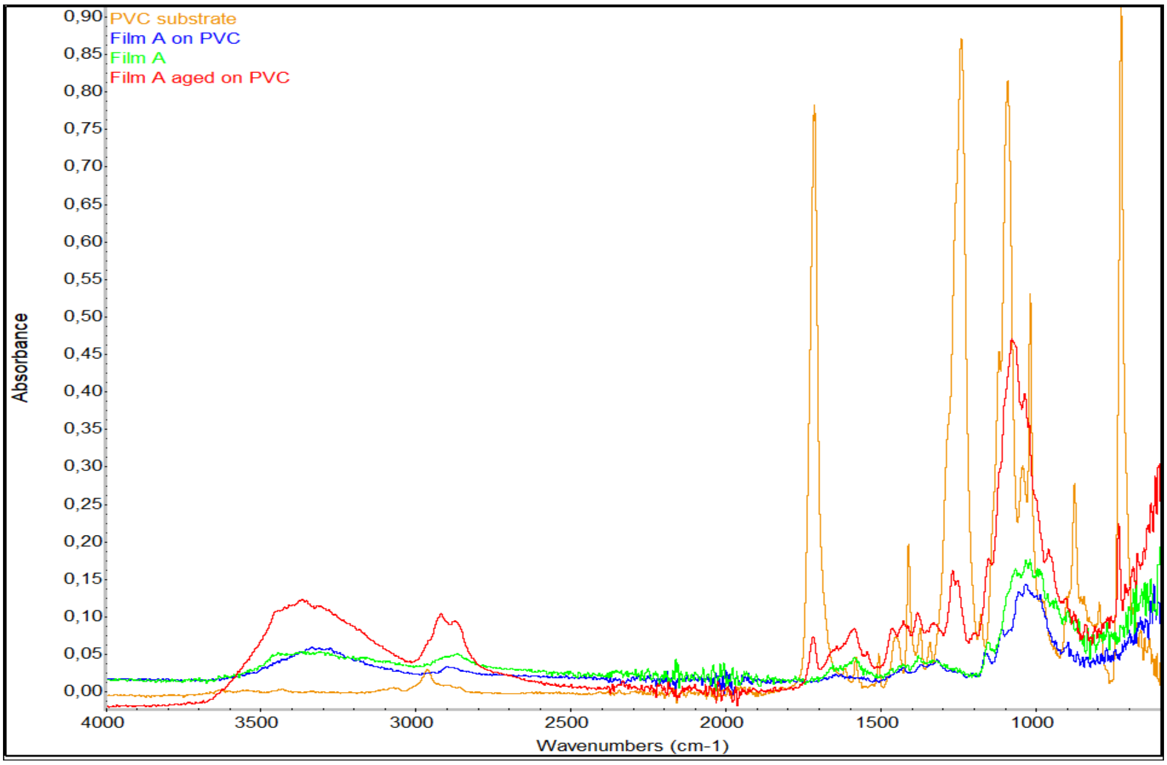

Figure S1. Comparison of FTIR-ATR spectra collected from Film A unaged and aged, both applied on the p-PVC sheet, Film A as a single component, and p-PVC sheet.

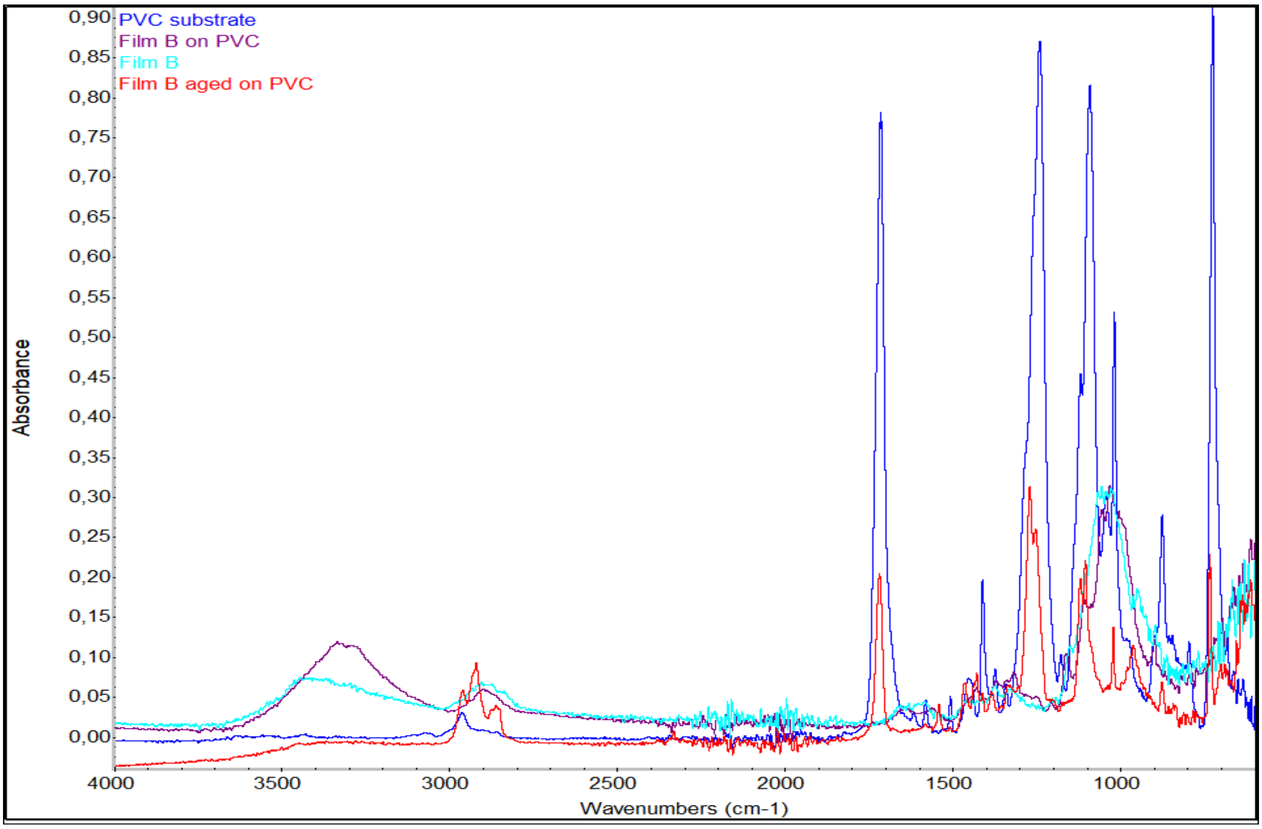

Figure S2. Comparison of FTIR-ATR spectra collected from Film B unaged and aged, both applied on the p-PVC sheet, Film B as a single component, and p-PVC sheet.

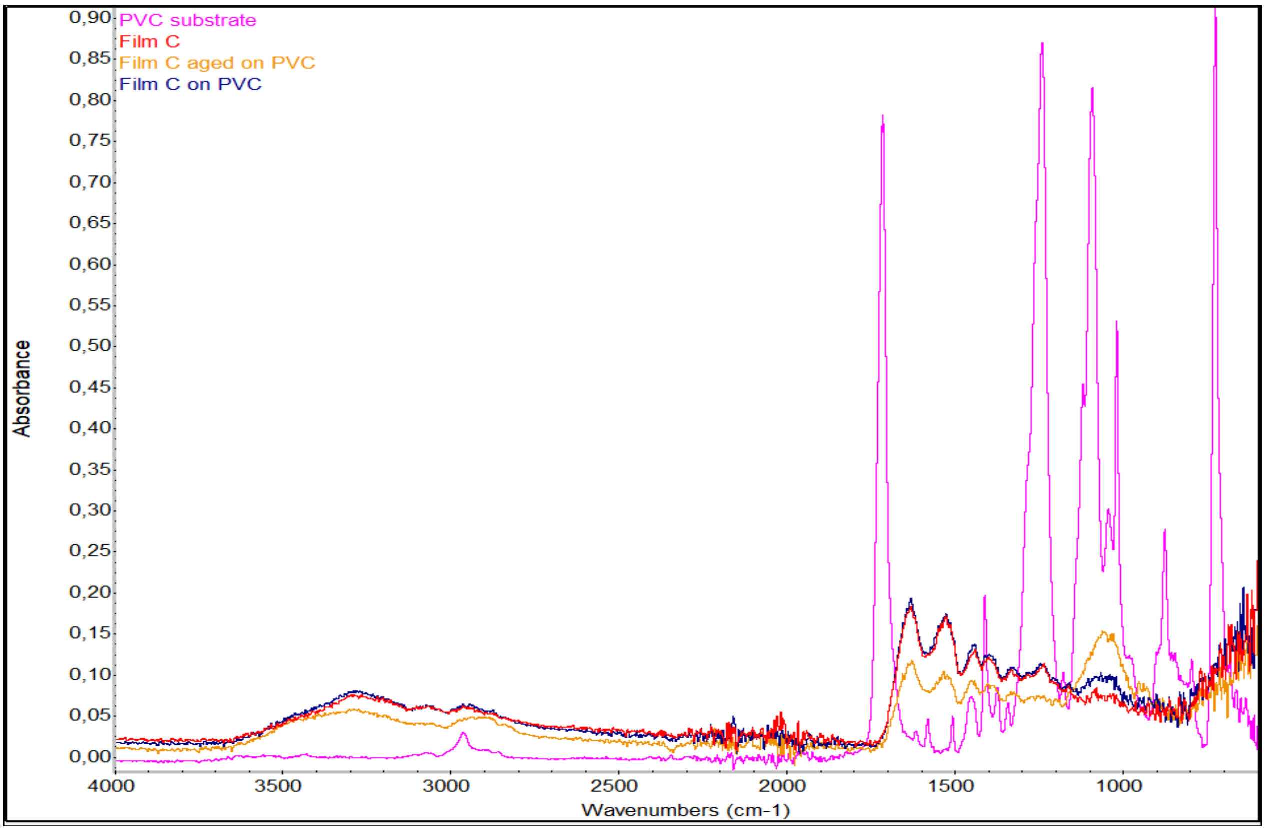

Figure S3. Comparison of FTIR-ATR spectra collected from Film C unaged and aged, both applied on the p-PVC sheet, Film C as a single component, and p-PVC sheet.

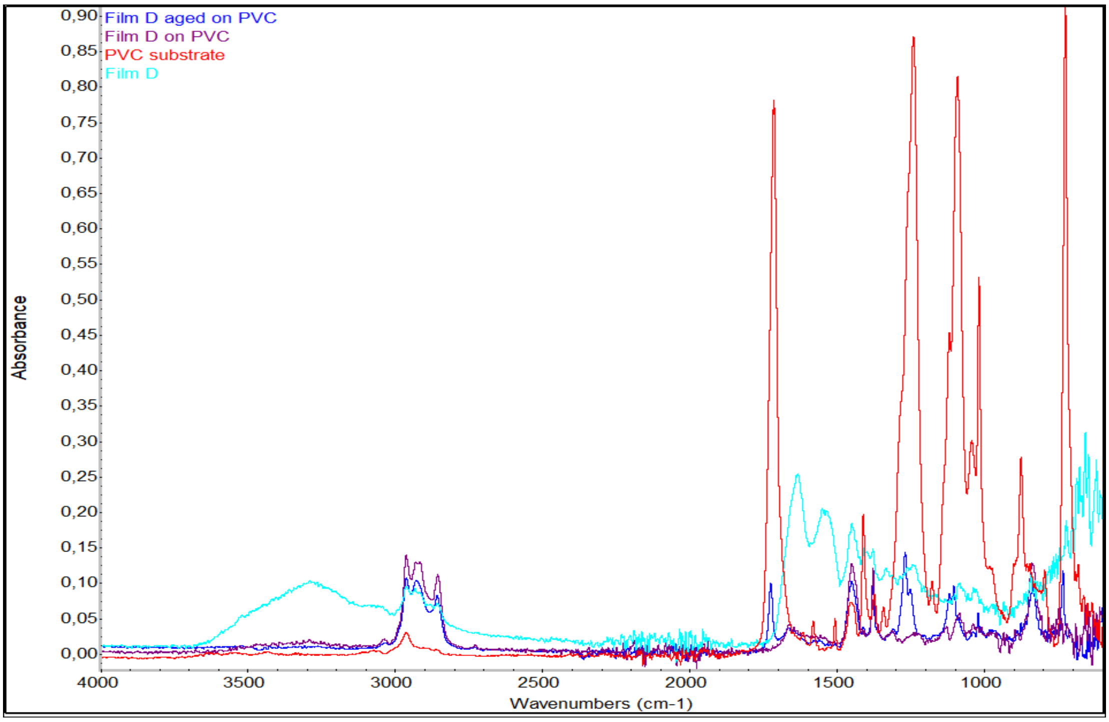

Figure S4. Comparison of FTIR-ATR spectra collected from Film D unaged and aged, both applied on the p-PVC sheet, Film D as a single component, and p-PVC sheet.

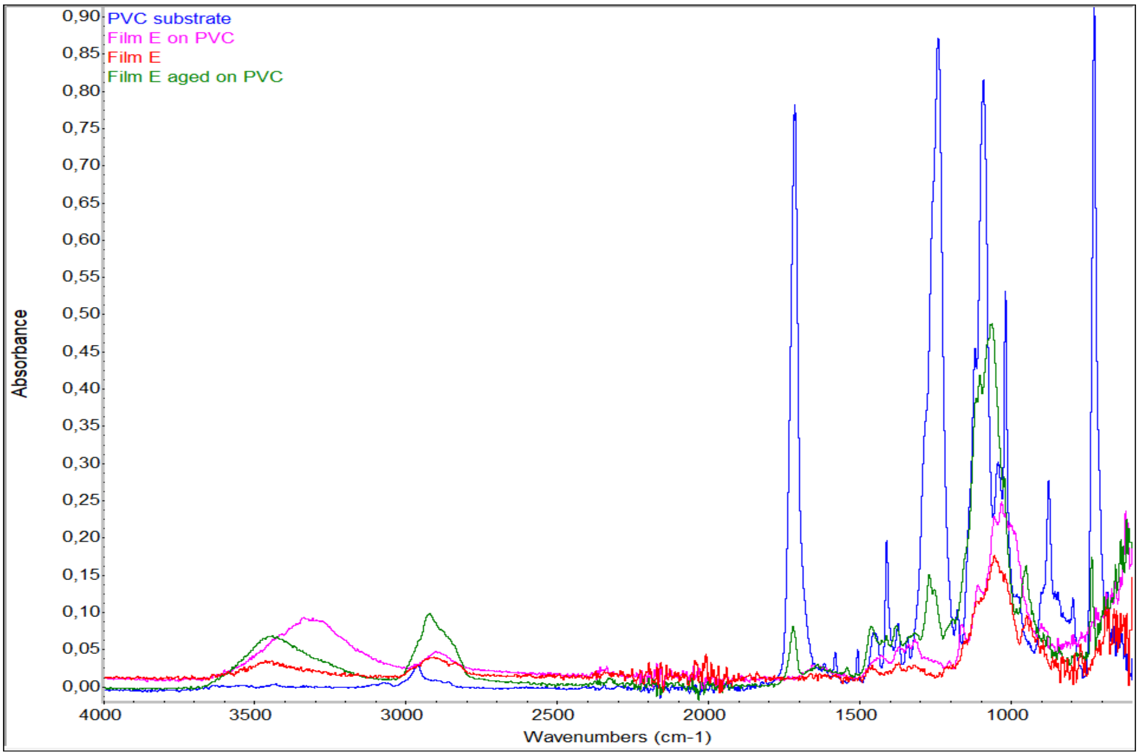

Figure S5. Comparison of FTIR-ATR spectra collected from Film E unaged and aged, both applied on the p-PVC sheet, Film E as a single component, and p-PVC sheet.

Disclaimer/Publisher's Note: The statements, opinions and data contained in all publications are solely those of the individual author(s) and contributor(s) and not of MDPI and/or the editor(s). MDPI and/or the editor(s) disclaim responsibility for any injury to

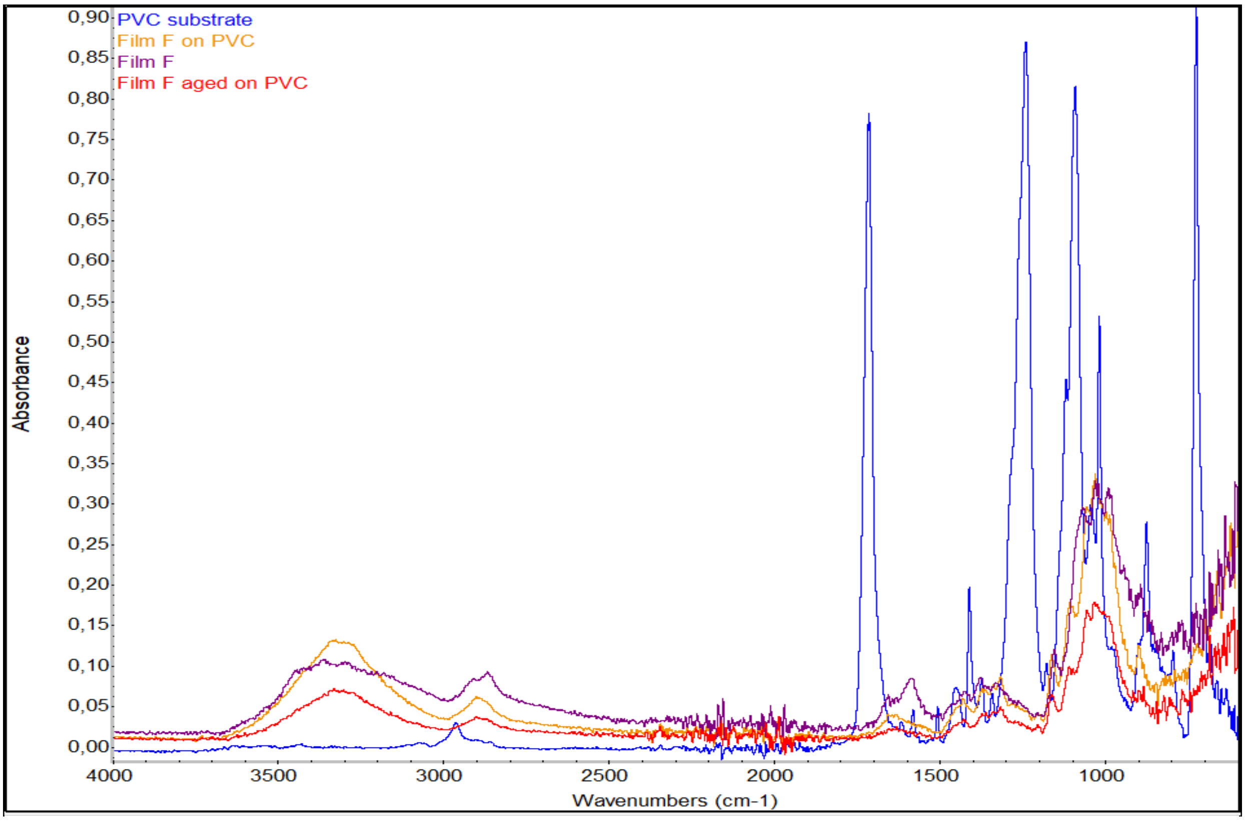

Figure S6. Comparison of FTIR-ATR spectra collected from Film F unaged and aged, both applied on the p-PVC sheet, Film F as a single component, and p-PVC sheet.

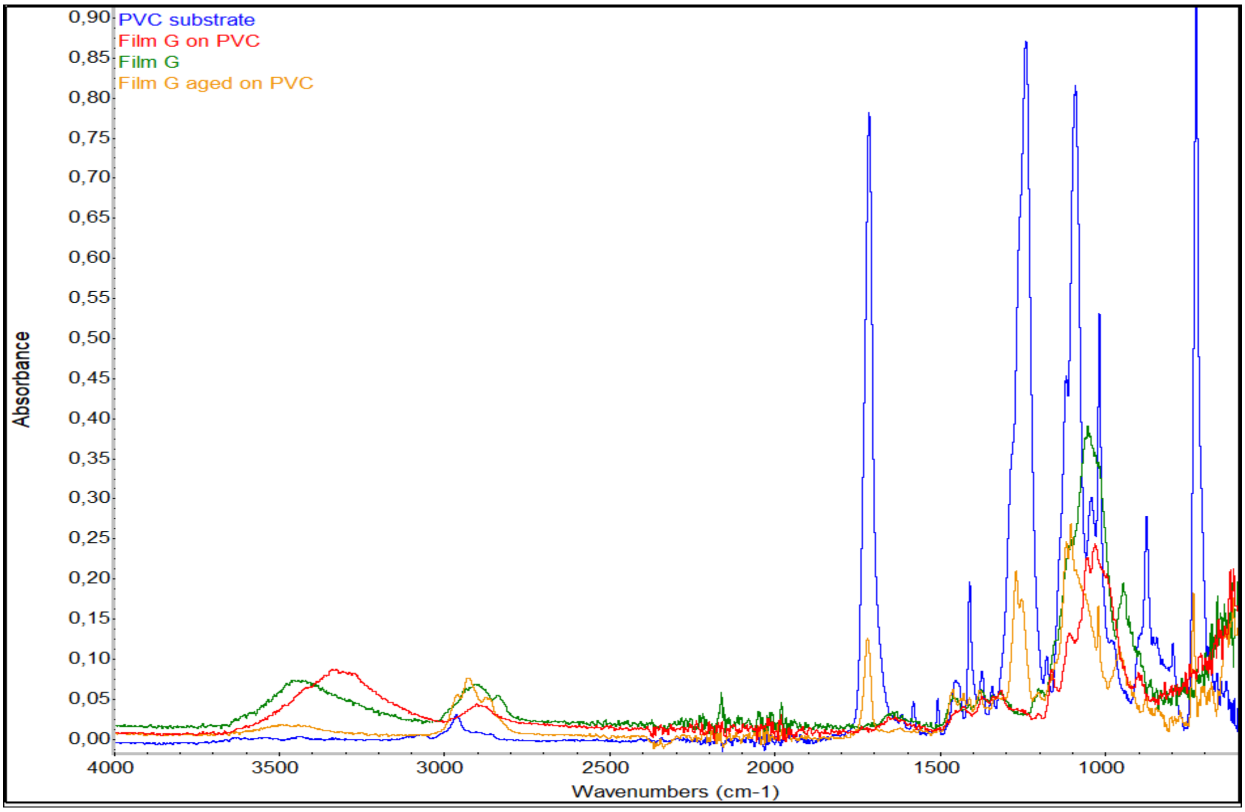

Figure S7. Comparison of FTIR-ATR spectra collected from Film G unaged and aged, both applied on the p-PVC sheet, Film G as a single component, and p-PVC sheet.

people or property resulting from any ideas, methods, instructions or products referred to in the content.
